# Artificial intelligence applied to omics data in liver diseases: Enhancing clinical predictions

**DOI:** 10.3389/frai.2022.1050439

**Published:** 2022-11-15

**Authors:** Cristina Baciu, Cherry Xu, Mouaid Alim, Khairunnadiya Prayitno, Mamatha Bhat

**Affiliations:** ^1^Ajmera Transplant Program, University Health Network, Toronto, ON, Canada; ^2^Faculty of Health Sciences, McMaster University, Hamilton, ON, Canada; ^3^Departments of Computer Science and Cell and System Biology, University of Toronto, Toronto, ON, Canada; ^4^Division of Gastroenterology and Hepatology, University Health Network and University of Toronto, Toronto, ON, Canada; ^5^Toronto General Research Institute, University Health Network, Toronto, ON, Canada

**Keywords:** artificial intelligence, machine learning, omics data, liver disease, clinical outcome prediction

## Abstract

Rapid development of biotechnology has led to the generation of vast amounts of multi-omics data, necessitating the advancement of bioinformatics and artificial intelligence to enable computational modeling to diagnose and predict clinical outcome. Both conventional machine learning and new deep learning algorithms screen existing data unbiasedly to uncover patterns and create models that can be valuable in informing clinical decisions. We summarized published literature on the use of AI models trained on omics datasets, with and without clinical data, to diagnose, risk-stratify, and predict survivability of patients with non-malignant liver diseases. A total of 20 different models were tested in selected studies. Generally, the addition of omics data to regular clinical parameters or individual biomarkers improved the AI model performance. For instance, using NAFLD fibrosis score to distinguish F0-F2 from F3-F4 fibrotic stages, the area under the curve (AUC) was 0.87. When integrating metabolomic data by a GMLVQ model, the AUC drastically improved to 0.99. The use of RF on multi-omics and clinical data in another study to predict progression of NAFLD to NASH resulted in an AUC of 0.84, compared to 0.82 when using clinical data only. A comparison of RF, SVM and kNN models on genomics data to classify immune tolerant phase in chronic hepatitis B resulted in AUC of 0.8793–0.8838 compared to 0.6759–0.7276 when using various serum biomarkers. Overall, the integration of omics was shown to improve prediction performance compared to models built only on clinical parameters, indicating a potential use for personalized medicine in clinical setting.

## Introduction

Artificial Intelligence (AI)-based tools are being increasingly used for the early diagnosis, prediction of disease progression or survival also increases. The rapid development of biotechnology has revolutionized biomedical research by providing high-throughput molecular data, alongside clinical information collected in health records (Spann et al., 2020; Castañé et al., 2021). As part of AI, both conventional machine-learning (ML) and new deep-learning (DL) algorithms use a data-driven approach that efficiently screens high-throughput data in an unbiased manner, uncovering hidden patterns and generating results which may inform clinical decisions (Chen and Asch, 2017; Spann et al., 2020). Early detection of liver disease can be difficult with conventional methods and AI offers a more comprehensive approach wherein patterns in both clinical and molecular data could be leveraged for insight into diagnostic and prognostic predictions.

Conventional ML is the branch of AI that focuses on processing and learning from data to improve the accuracy and performance of the machine without being explicitly programmed (Bini, 2018). The algorithms are self-learning tools that can be used for efficient and accurate prediction, classification, and resource allocation. They are optimized on a training set and their performance are evaluated on a test set. Supervised ML algorithms [k-nearest neighbor (kNN), linear regression, logistic regression (LR), support vectors machines (SVM), naïve bayes (NB), decision trees and random forests (RF)] work on labeled data where each input is mapped to a corresponding label. On the contrary, unsupervised algorithms (k-means clustering, anomaly detection, association rules, and dimensionality reduction algorithms) use unlabeled input data with the scope of the creation of labels (Dhall et al., 2020).

### Deep learning and neural networks

Neural networks (NNs) or artificial neural networks (ANNs) are the subdivision of ML that focuses on emulating the neurons of the brain. They process signals from an external stimulus and determine the correct downstream course of action. NNs are made up of three layers. Initially, the neurons of the input layer receive signal from the environment. This input is passed on to the neurons in the hidden layer that perform the necessary calculations. The resulting message is then passed along to the output layer of neurons. One application of NNs, deep learning (DL), utilizes a plethora of hidden layers or abstraction units, where each unit performs non-linear operations to process the data (LeCun et al., 2015). Furthermore, the ability of DL to simultaneously accept different forms of input data elevates its performance above traditional machine learning (Esteva et al., 2019). This has led to an increase in DL applications in the field of medicine, such as the use of convolutional neural networks (CNN) for medical imaging and classification.

In liver disease, AI has been applied to various omics data, including genomics, transcriptomics, proteomics, metabolomics and microbiomics for early diagnosis, biomarker discovery, as well as prediction of disease progression and survival. This mini review aims to summarize existing literature on AI models built using a combination of high-throughput omics datasets and/or of clinical variables for the early identification, stratification, and survival prediction of non-malignant liver diseases.

### Literature search

A PubMed search was conducted to retrieve studies published up to Nov 18^th^, 2021. The inclusion criteria were defined as original papers on human participants in a non-clinical trial setting. We screened and selected eligible studies based on their title and abstract. Our search initially identified 940 papers. After applying the exclusion criteria we selected 20 studies for the final review. The exclusion criteria consisted of following: no liver disease (*n* = 163) or liver related (*n* = 104), animal studies (*n* = 67), clinical trials (*n* = 28), no ML (*n* = 32), non-articles (*n* = 16), non-English (*n* = 8), malignancy (*n* = 143), radiomics (*n* = 114), treatment related (*n* = 96,) reviews (*n* = 57), no clinical or omics data (*n* = 77), editorial/reflection/environmental/language processing or epidemiological studies (*n* = 15). The extracted information included: number of study participants and their clinical data, study objective, type of liver disease, omics type, AI model and performance metrics. The objectives, AI algorithms used, sample and clinical information on the studies from this mini review, classified by the liver diseases, were included in Table 1. The studies that performed comparison of AI algorithms and their performance to models using only clinical parameters, biomarkers or single features were summarized in Table 2.

**Table 1:** Summary of the studies included in this review classified by the liver diseases, with details on the objectives, ML algorithm used, sample and clinical information.

**Author/Objectives/ML algorithm**	**Sample size**	**Clinical information**
**Non-alcoholic fatty liver disease/Non-alcoholic Steatohepatitis (NAFLD/NASH)**		
Atabaki-Pasdar et al. (2020) *To predict and aid in early diagnosis of NAFLD /* RF	European-ancestry adults diagnosed with T2D (*n* = 795) or at high risk of developing the disease (*n* = 2,234)	Liver enzymes and other serological biomarkers, Anthropometry, Measures of beta-cell function, Insulin sensitivity, and Lifestyle
Moolla et al. (2020) *To distinguish various stages of NAFLD using urinary steroid metabolome /* GMLVQ	275 subjects (*n* = 121 with biopsy-proven NAFLD, *n* = 48 with alcohol-related cirrhosis and *n* = 106 controls)	Urinary steroid metabolites and creatinine
Masarone et al. (2021) *To differentiate simple steatosis, steatohepatitis, and cirrhosis in NAFLD patients /* PLS-DA	Training: controls (*n* = 69) and patients [steatosis (*n* = 78), NASH (*n* = 23), NASH-cirrhosis (*n* = 15), HCV-cirrhosis (*n* = 8), cryptogenic cirrhosis, (*n* = 20)]. Validation: 44 controls and patients [steatosis (*n* = 34), NASH (*n* = 10) and NASH-cirrhosis, (*n* = 6)]	Age, Sex ratio, BMI, serum cholesterol concentration (both HDL an LDL), triglycerides, ferritin and GGT resulted among the patients. Blood insulin concentration, homeostatic model assessment (HOMA), prevalence of hypertension, type 2 diabetes mellitus, metabolic syndrome and mean liver stiffness
Chiappini et al. (2017) *To diagnose NAFLD using lipid profiling /* RF	Liver biopsies including normal livers as controls (*n* = 7), NAFL (*n* = 39) and NASH (*n* = 15)	N/A
Perakakis et al. (2019) *To diagnose NASH and liver fibrosis /* SVM, kNN, RF	Healthy subjects as controls (*n* = 49) and patients with biopsy-proven of NAFL (*n* = 15) and NASH (*n* = 16)	adiponectin, leptin, activin A, follistatin and triglycerides
Wang T. et al. (2021) *To predict and determine the stages of NAFLD /* GBM, RF	Samples from patients with NAFL (*n* = 39), NASH (*n* = 39), fibrosis (*n* = 15), cirrhosis (*n* = 14), and healthy controls (*n* = 120)	Age, BMI, Sex
Oh et al. (2020) *To develop a non-invasive diagnostic tool for cirrhosis, advanced fibrosis, and NAFLD /* RF	163 well-characterized participants with diagnoses that covered the spectrum of non-alcoholic fatty liver disease (NAFLD), including non-NAFLD controls (*n* = 54), NAFLD-cirrhosis patients (*n* = 27) as well as their first-degree relatives.	Age, Sex, Ethnicity, BMI, Liver biopsy, Type 2 diabetes, AST, ALT, Alk P, GGT, Total Bilirubin, Direct Bilirubin, Albumin, Glucose, Hemoglobin A1c, Insulin, Triglycerides, Total cholesterol, HDL-cholesterol, LDL-cholesterol, Platelet count, Prothrombin time, INR, Ferritin, MRI proton density fat fraction (MRI-PDFF) %, magnetic resonance, elastography (MRE) (kPa)
Luo et al. (2021) *To diagnose NASH and differentiate between fibrosis stages in NASH patients /* Elastic-Net	Samples from patients with NASH (*n* = 113): stage 0 (*n* = 13), stage 1 (*n* = 19), stage 2 (*n* = 36), stage 3 (*n* = 23), stage 4 (*n* = 22)	Liver biopsy images, serum samples, Age, gender, BMI, ALT, AST, Platelet count, T2DM, NAFLD activity score
**Viral hepatitis (HBV/HCV)**		
Mueller-Breckenridge et al. (2019) *To classify HBV e antigen status /* RF	Dataset A: plasma samples from a Western European cohort (*n* = 182) Dataset B: plasma samples from a Chinese cohort (*n* = 207)	Age, Sex, Duration of infection, Serum samples, Paired Liver biopsy, HBV DNA load, AST, ALT, HBeAg status, Treatment (naïve/established), Serum qualitative HBsAg, anti-HBs, HBeAg, anti-HBeAg, HBV viral load
Wang M. et al. (2021) *To differentiate patients with chronic HBV from the immune tolerant stage /* kNN, SVM, RF	290 liver biopsies from HBeAg positive patients divided into training group (*n* = 148) and validation group (*n* = 142)	Serum samples; HBV serological biomarkers (HBsAg, HBs antibody, HBeAg, HBe antibody, and hepatitis B core antibody)
Wu et al. (2021) *To predict HBV integration sites in promoting malignant transformation /* CNN	HBV integration sites: Positive samples datasets training (*n* = 2,902) and testing (*n* = 1,264) Negative samples datasets training (*n* = 5,804) and testing (*n* = 2,528)	N/A
Huang et al. (2007) *To predict the risk of developing cirrhosis in chronic HCV patients /* NB	HCV patients: Training: *n* = 420 Validation: *n* = 154	Demographics (date of birth, sex, and ethnicity), virological features (HCV genotype and serum HCV RNA level), and liver histology.
Zhou et al. (2017) *To analyze correlations among inflammation grades, gene expressions and clinical parameters, and prediction of inflammation grades /* RF, SVM, kNN	Chronic HBV samples (*n* = 122)	Sex, Age, serum ALT, AST and HBV-DNA
Lara et al. (2014) *To differentiate between fast and slow rate of fibrosis among chronic HCV patients /* LP, BN	Transplant recipient with HCV (*n* = 22) Non-transplanted immunocompetent patients with HCV (*n* = 20)	Rate of fibrosis progression, Transplant status
Cao et al. (2013) *To differentiate chronic HBV patients with cirrhosis from non-cirrhosis controls /* LC-NB, LC-MLP, LC-SVM, LC-DT, LC-CART	162 serum samples including cirrhosis patients with chronic HBV (*n* = 44), chronic HBV(*n* = 46), and healthy individuals (*n* = 72)	Age, Sex
**Drug-induced liver injury**		
Moore et al. (2021) *To predict the risk of developing drug-induced liver injury /* MARS	271 patients (33 chronic vs. 238 acute DILI)	Dili status (chronic or acute)
**Primary sclerosing cholangitis**		
Iwasawa et al. (2018) *To distinguish salivary microbial communities of PSC patients from healthy controls /* RF	Pediatric-onset PSC patients (*n* = 24), age-matched ulcerative colitis patients (*n* = 16), and healthy controls (*n* = 24)	Age at onset, Age at diagnosis, PSC phenotype, Type of IBD, Biochemical data, Medication
Mousa et al. (2021) *To predict 5-year hepatic decompensation risk in PSC patients /* GBM	Patients with PSC (*n* = 400) and controls (*n* = 302) in the discovery cohort. Patients with PSC (*n* = 108) in the validation cohort	Age, Sex, Bile acid profiles, IBD status, PSC subtype including small duct disease and overlap with autoimmune hepatitis (AIH), UDCA treatment and progression to clinical endpoints
**Liver echinococcosis**		
Peng et al. (2021) *To predict and diagnose liver echinococcosis /* RF	6 liver echinococcosis patients	N/A
**Liver diseases - general**		
Lin et al. (2011) *To classify patients with hepatocellular carcinoma, cirrhosis and hepatitis /* F-SVM	Plasma samples from patients with: HCC (*n* = 28), chronic HBV (*n* = 26), cirrhosis (*n* = 28) and healthy controls (*n* = 30)	Ultrasonography and computerized tomography (CT) imaging, Physical and biochemical tests (AFP, carcinoembryonic antigen, HBV surface antigen, antibody to hepatitis surface antigen, glutamic pyruvic transaminase, glutamic oxalacetic transaminase, albumin, globin)

ALP, Alkaline Phosphatase; ALT, Alanine Transaminase; aNN, Artificial Neural Networks; APRI, Aspartate Transaminase to Platelet Ratio Index; AUPR, Area Under the Precision Recall Curve; BN, Bayesian Networks; CART, Classification and Regression Tree; CI, confidence interval; CNN, Convolutional Neural Network; DC, Decision Tree; DL, Deep Learning; F-SVM, Feature Support Vector Machine; FIB-4, Fibrosis-4; GBM, Gradient-Boosting Machine; GMLVQ, Generalized Matrix Learning Vector Quantization; kNN, K-Nearest Neighbor; LC, Liver Cirrhosis; LDA, Linear Discriminant Analysis; LP, Linear Projection; LR, Logistic Regression; MARS, Multivariate Adaptive Regression Splines; MDR, Multifactor Dimensionality Reduction; ML, Machine Learning; MP, Multilayered Perceptron; NAFLD, non-alcoholic fatty liver disease; NASH, non-alcoholic steatohepatitis; NB, Naïve Bayes; OR, odds ratio; PLS-DA, Partial Least Square Discriminant Analysis; PSC, Primary Sclerosing Cholangitis; RF, Random Forest; SVM, Support Vector Machine.

**Table 2:** Selected studies that performed comparison of ML methods and their performance to models using only clinical parameters, biomarkers or single features.

**omics type**	**Author and study objective**	**ML method and model performance on omics data**		**Comparison with ML on non-omics or with non-ML models**
**Non-alcoholic fatty liver disease/Non-alcoholic steatohepatitis**				
**Multi-omics (genomics, metabolomics, transcriptomics, proteomics)**	Atabaki-Pasdar et al. (2020) *To predict and aid in early diagnosis of NAFLD*.	RF	On multi-omics: AUROC 0.82 (95% CI: 0.80–0.84, *p* < 0.001) on multi-omics + clinical parameters: AUROC 0.84 (95% CI: 0.82, 0.86, *p* < 0.001)	RF on clinical parameters: AUROC 0.82 (95% CI: 0.81, 0.83, *p* < 0.001) fatty liver index: AUROC 0.78 (95% CI: 0.76–0.80, *p* < 0.001) hepatic steatosis index: AUROC 0.76 NAFLD-liver fat score: AUROC: 0.76 (95% CI: 0.75–0.78, *p* < 0.001)
**Metabolomics**	Moolla et al. (2020) *To distinguish various stages of NAFLD using urinary steroid metabolome*.	GMLVQ	To distinguish between: - control and advanced fibrotic NAFLD: AUC ROC 0.99 (95% CI: 0.98–0.99) - control and NAFLD cirrhosis: AUC ROC 1.0 (95% CI: 1.0–1.0) - NAFLD cirrhosis and alcohol-related cirrhosis: AUC ROC 0.83 (95% CI: 0.81–0.85)	To distinguish F0-F2 from F3-F4: - NAFLD fibrosis score: AUC ROC 0.87 (95% CI: 0.86–0.88) - FIB-4 index: AUC ROC 0.91 (0.89–0.92)
	Perakakis et al. (2019) *To diagnose NASH and liver fibrosis*.	SVM (non-linear)	On lipids: accuracy 0.88 On lipids + glycans: accuracy 0.82 On lipids + hormones: accuracy 0.87 On lipids + hormones + glycans: accuracy 0.86	SVM accuracy on glycans: 0.54, on hormones: 0.55 kNN accuracy on glycans: 0.45, on hormones: 0.49 RF accuracy on glycans: 0.49, on hormones: 0.54
		kNN	On lipids: accuracy 0.65 On lipids + glycans: accuracy 0.67 On lipids + hormones: accuracy 0.64 On lipids + hormones + glycans: accuracy 0.63	
		RF	On lipids: accuracy 0.86 On lipids + glycans: accuracy 0.86 On lipids + hormones: accuracy 0.80 On lipids + hormones + glycans: accuracy 0.83	
**Viral hepatitis (HBV/HCV)**				
**Genomics**	Wang M. et al. (2021) *To differentiate patients with chronic HBV from the immune tolerant stage*.	kNN	AUC 0.88 (accuracy 0.90, sensitivity 0.82, specificity 0.94)	HBsAg index: AUC 0.6759 (accuracy 0.6922, sensitivity 0.7447, specificity 0.6744) ALT index: AUC 0.6806 (accuracy 0.5845, sensitivity 0.8958, specificity 0.4255) APRI index: AUC 0.7033 (accuracy 0.6917, sensitivity 0.7143, specificity 0.6813) FIB-4 index: AUC 0.7276 (accuracy 0.6617, sensitivity 0.6905, specificity 0.6484)
		SVM	AUC 0.8838 (accuracy 0.90, sensitivity 0.83, specificity 0.93)	
		RF	AUC 0.8793 (accuracy 0.90, sensitivity 0.81, specificity 0.94)	
	Wu et al. (2021) *To predict HBV integration sites in promoting malignant transformation*.	CNN	on HBV integration sequence + repeats: AUROC 0.84, AUPR 0.75 on HBV integration sequence + TCGA Pan-Cancer peaks: AUROC 0.94, AUPR 0.93	CNN on HBV integration sequences: AUROC 0.64, AUPR 0.55
	Huang et al. (2007) *To predict the risk of developing cirrhosis in chronic HCV patients*.	NB	On cirrhosis risk score: AUC 0.73 (95% CI: 0.56–0.89, *P* < 0.001) On cirrhosis risk score + clinical parameters: AUC 0.76 (95% CI: 0.60–0.92, *P* < 0.001)	NB on clinical parameters: AUC 0.53 (95% CI: 0.35–0.72, *P* = 0.36)
**Transcriptomics**	Zhou et al. (2017) *To analyze correlations among inflammation grades, gene expressions and clinical parameters, and prediction of inflammation grades*.	RF	On gene panel: AUC 0.80 (95% CI: 0.69–0.89) (accuracy 0.70, sensitivity 0.72, specificity 0.68) On gene panel + clinical parameters: AUC 0.88 (95% CI: 0.77–0.93) (accuracy 0.73, sensitivity 0.77, specificity 0.68)	RF on clinical parameters: AUC 0.78 (95% CI: 0.65–0.86) (accuracy 0.69, sensitivity 0.77, specificity 0.61); SVM on clinical parameters: AUC 0.73 (95% CI: 0.58–0.81) (accuracy 0.67, sensitivity 0.72, specificity 0.61); kNN on clinical parameters: AUC 0.73 (95% CI: 0.62–0.84) (accuracy 0.69, sensitivity 0.80, specificity 0.58)
		SVM	On gene panel: AUC 0.75 (95% CI: 0.58–0.82) (accuracy 0.67, sensitivity 0.67, specificity 0.66) On gene panel + clinical parameters: AUC 0.71 (95% CI: 0.58–0.81) (accuracy 0.65, sensitivity 0.65, specificity 0.66)	
		kNN	On gene panel: AUC 0.72 (95% CI: 0.65–0.86) (accuracy 0.72, sensitivity 0.82, specificity 0.69) On gene panel + clinical parameters: AUC 0.8075 (95% CI: 0.71–0.90) (accuracy 0.77, sensitivity 0.89, specificity 0.62)	
**Drug-induced liver injury**				
**Genomics**	Moore et al. (2021) *To predict the risk of developing drug-induced liver injury*.	MARS	on SNP interactions: OR 4.19 (95% CI: 1.36–11.74, *p* = 0.008)	MARS on individual SNPs: OR 3.28/1.49 (95% CI: 1.57–7.09/0.72–3.14, *p* = 0.002/0.282) MDR on individual SNP: 3.01 (95% CI: 1.44–6.43, *p* = 0.004)
**Primary sclerosing cholangitis (PSC)**				
**Metabolomics**	Mousa et al. (2021) *To predict 5-year hepatic decompensation risk in PSC patients*.	GBM	PSC-Bile Acid Profile score (validation cohort): AUC 0.86 (95% CI: 0.74–0.96)	GBM on PSC Risk Estimate Tool: AUC 0.94 (95% CI: 0.87–0.98) Using cox proportional hazard models: - Bilirubin score: AUC 0.77 - ALP score: AUC 0.70

ALT, Alanine Transaminase; APRI, Aspartate Transaminase to Platelet Ratio Index; AUPR, Area Under the Precision Recall Curve; CI, confidence interval; CNN, Convolutional Neural Network; FIB-4, Fibrosis-4; GBM, Gradient-Boosting Machine; GMLVQ, Generalized Matrix Learning Vector Quantization; kNN, K-Nearest Neighbor; MARS, Multivariate Adaptive Regression Splines; NB, Naïve Bayes; OR, odds ratio; RF, Random Forest; SVM, Support Vector Machine.

## Discussion

### AI for early diagnosis and progression of NAFLD/NASH

NAFLD/NASH was the most studied disease, being reported in eight out of the 20 included studies (Chiappini et al., 2017; Perakakis et al., 2019; Atabaki-Pasdar et al., 2020; Moolla et al., 2020; Oh et al., 2020; Masarone et al., 2021; Wang T. et al., 2021). Using multi-omics, two studies used random forest (RF) models for early diagnosis of NAFLD and prediction of the progression from NAFLD to NASH (Atabaki-Pasdar et al., 2020; Oh et al., 2020). Atabaki-Pasdar et al. conducted a multicenter prospective cohort study of 3,028 adults, who were recently diagnosed with or found at risk of developing diabetes, to develop a non-invasive NAFLD prediction tool (Atabaki-Pasdar et al., 2020). Multi-omics data including genomics, transcriptomics, proteomics and metabolomics, along with clinical parameters, were incorporated into a web interface after model validation. The RF model incorporating omics with clinical parameters yielded a cross-validated area under the receiver operating characteristic curve (AUROC) of 0.84 (95% CI 0.82, 0.86, *p* < 0.001) (Atabaki-Pasdar et al., 2020) that was superior than the model incorporating only clinical information (AUROC) of 0.82 (95% CI 0.81, 0.83, *p* < 0.001). Oh et al. employed a combination of metagenomics and metabolomics, along with typical clinical variables, to predict the progression of NAFLD to advanced fibrosis and cirrhosis (Oh et al., 2020). Their RF models distinguishes cirrhosis from NAFLD and mild or moderate fibrosis (AUROC of 0.85 and 0.84, respectively) with an improved accuracy upon incorporating serum AST level (AUROC of 0.94 and 0.91, respectively) (Oh et al., 2020). Another four studies utilized metabolomics for various purposes, including the prediction of NAFLD, diagnosis of NASH, and differentiation of NAFLD stages (Chiappini et al., 2017; Perakakis et al., 2019; Moolla et al., 2020; Masarone et al., 2021). The models included decision tree, naïve bayes (NB), random forest (RF), artificial neural network (ANN), partial least square discriminant analysis (PLS-DA), linear discriminant analysis (LDA), DL, and logistic regression (LR). Chiappini et al. used liver biopsies and lipid profiling to develop an RF model aimed to differentiate between NASH and different stages of NAFL (Chiappini et al., 2017). Their RF model based on a 32-lipid signature, associated with dysregulation in fatty acid synthesis pathway, showed 100% sensitivity and specificity, AUC = 1. Uniquely, Perakakis et al. conducted a proof-of-concept study which used SVM, kNN, linear PLS-DA and RF models to diagnose NASH and liver fibrosis based on single or combination of lipids, glycans and biochemical profile (Perakakis et al., 2019). The non-linear SVM with selection of biomolecules were the best predictive ML models for NASH vs. NAFL vs. Healthy groups, with AUC varying from 0.77 to 0.99, depending on the selected variables (see Table 2 for more details). Notably, Masarone et al. used the urinary steroid metabolome to construct both ML and DL models for differentiating between different stages, e.g., simple steatosis, steatohepatitis and cirrhosis in NAFLD patients (Masarone et al., 2021). The models include decision tree, naïve Bayes (NB), random forest (RF), artificial neural network (ANN), partial least square discriminant analysis (PLS-DA), linear discriminant analysis (LDA), DL, and logistic regression (LR), with PLS-DA showing best performance. Depending on which two-group comparison was performed to differentiate between various conditions (NAFLD and Controls, NAFLD and NASH or NASH and cirrhosis), the accuracy of the PLS-DA models was very high, ranging from 0.991 to 0.999 and quality of the prediction indicated by R^2^, from 0.928 to 0.968. Similarly, Moolla et al. also differentiated various stages using a ML-based generalized matrix learning vector quantization (GMLVQ) analysis on urinary metabolomic data (Moolla et al., 2020). All these models were reported to have an accuracy >0.80 (Perakakis et al., 2019; Moolla et al., 2020; Masarone et al., 2021). A proteomics study used an elastic-net algorithm to diagnose NASH and differentiate between fibrosis stages in NASH patients and built models based on a four- or 12-protein classifier (AUROC of 0.74 and 0.83, respectively) (Luo et al., 2021).

### AI applied to viral hepatitis

A total of seven studies reported on the omics profiling of viral hepatitis patients, specifically hepatitis B virus (HBV) and hepatitis C virus (HCV) infections (Huang et al., 2007; Cao et al., 2013; Lara et al., 2014; Zhou et al., 2017; Mueller-Breckenridge et al., 2019; Wang M. et al., 2021; Wu et al., 2021). Of these, five studies used genomics (Huang et al., 2007; Zhou et al., 2017; Mueller-Breckenridge et al., 2019; Wang M. et al., 2021; Wu et al., 2021), one study utilized transcriptomics (Lara et al., 2014), and one study generated proteomics data (Cao et al., 2013). HBV is a major risk factor for developing liver fibrosis, cirrhosis, and hepatocellular carcinoma (HCC) (Mueller-Breckenridge et al., 2019). Hepatitis B e antigen (HBeAg) is a secreted protein of HBV with a high sequence conservation and seroconversion due to HBeAg represents a biomarker in infected individuals (Mueller-Breckenridge et al., 2019). Mueller-Breckenridge et al. used the RF method to classify HBeAg status of patients with chronic HBV infection (Mueller-Breckenridge et al., 2019). The highest-ranking variables contributing to the model are known pre-core and basal core promotor mutants (n1896GA, n1934AT, n1753TC). Similarly, Wang et al. used the HBeAg biomarker and enrolled HBeAg positive patients from whom liver biopsies were taken (Wang M. et al., 2021). Using three ML models, i.e., kNN, SVM, and RF, the study aimed to differentiate the immune tolerant phase from chronic HBV. All predictive ML models using genomics (whole HBV sequencing data and viral quasispecies), showed higher AUC values (validation group AUC of 0.8838, 0.8801, and 0.8793 for SVM, kNN, and RF, respectively) compared to models commonly used for liver fibrosis, based on HBsAg, ALT, APRI, and FIB-4 (validation group AUC of 0.6759, 0.6806, 0.7033 and 0.7276, respectively), indicating better classification (Wang M. et al., 2021) (Table 2). The authors suggested to use the best performing model for future diagnosis, rather than performing invasive liver biopsies. Wu et al. used Convolutional Neural Networks (CNN) to develop an attention-based deep learning model, DeepHBV (Wu et al., 2021). It effectively learns local genomic features and uses the information to predict HBV integration sites which promotes malignant transformation. In addition to DNA sequences near the HBV integration sites, the integration of additional genomic features, such as repeat and TCGA Pan-Cancer peaks, improved the AUROC from 0.64 to 0.84 and 0.94, respectively (Wu et al., 2021). Using this tool they revealed novel integration site for HBV. Zhou et al. used gene profiles and clinical variables to predict inflammation grades in chronic HBV patients (Zhou et al., 2017). A selection of genes along with clinical parameters showed effectiveness in predicting binary classifications of inflammation grade [AUROC of 0.88 (95% CI: 0.77–0.93)] and superior performance in comparison with the model that did not include clinical variables [AUROC of 0.78 (95% CI: 0.65–0.86)]. Although liver biopsy is the reference standard for evaluating cirrhosis, it is an invasive procedure. Cao et al. used proteomics data to differentiate HBV patients with cirrhosis from non-cirrhotic controls, by testing five machine learning models (NB), multilayered perceptron (MP), SVM, decision tree, and classification and regression tree (CART) on non-invasive serum biomarkers to diagnose cirrhosis. The best predictive model was based on NB, called LC-NB, with AUC of 0.977 (sensitivity: 86.4%, specificity: 96.9%, accuracy: 93.8%) (Cao et al., 2013), where LC stands for liver cirrhosis. By comparison, other ML models derived in this study obtained AUC varying from 0.73 (LC-CART) to 0.83 (LC-DT) to 0.85 (LC-SVM) to 0.97 (LC-MLP) and accuracy ranging from 0.85 to 0.86 to 0.90 to 0.98, respectively. ML (NB) was also applied for prediction of cirrhosis among Caucasian patients with chronic hepatitis C, by developing of Cirrhosis Risk Score (CRS) (Huang et al., 2007). The model that integrated a signature of seven SNPs with clinical variables presented superior performance (AUC of 0.76) vs. the model that did not include genomics data (AUC of 0.53). Lara et al. developed LP and BN models to differentiate between fast and slow rate of fibrosis progression (RFP) among chronic HCV patients (Lara et al., 2014). The LP model showed 95% accuracy of RFP-classification with a sensitivity of 82% and a specificity of 100%. The BN classifier models are learned separately from immunocompetent and liver transplant recipient datasets, reaching an accuracy of 86.4 and 85%, respectively (Lara et al., 2014).

### AI in drug-induced liver injury

The inclusion criteria set out to specifically predict DILI, as diagnosed by liver biopsy, was met by one study which utilized genomics data. The study by Moore et al. employed three ML algorithms, multivariate adaptive regression splines (MARS), multifactor dimensionality reduction (MDR), and LR, with the aim of investigating single-nucleotide polymorphisms (SNPs) (Moore et al., 2021). The effect of SNP-SNP interactions on DILI susceptibility as well as their ability to predict DILI chronicity were observed. Subsequently, an RF algorithm was used to validate the ability of the three models in identifying chronic DILI associated SNP interactions achieving sensitivity, specificity, accuracy and balanced accuracy scores of 42.4, 90.3, 86.3 and 66.4%, respectively. Ultimately, both the RF and LR algorithms were unsuccessful in more accurately identifying SNP-SNP interaction terms than individual SNPs while both MARS and MDR were successful. MARS identified interactions between SNPs rs6487213 and rs3785157 with an odds ratio score of 4.74 (95% CI: 2.14–10.39, *p* < 0.001), while MDR achieved an odds ratio of 4.19 (95% CI: 1.36–11.74, *p* = 0.008) for interactions between rs5417, rs7658048, and rs12453290.

### AI and primary sclerosing cholangitis

PSC accounted for two of the 20 final selected papers. One study involving metabolomic data from 400 PSC patients utilized both (univariate) Cox proportional hazard and (multivariable) GBM models (Mousa et al., 2021). They set out to predict the 5-year risk of hepatic decompensation in PSC patients. They developed a PSC bile acid profile (PSC-BAP) score able to sufficiently predict future hepatic decompensation events. For the final model with six covariates, the predictive performance was comparable in the training set (AUC of 0.95, 95%CI, 0.92–0.97) and in the validation cohort (AUC of 0.86, 95%CI, 0.74–0.96). Consequently, they elucidated a genuine potential for bile acid profiles as non-invasive biomarkers. Another study by Iwasawa et al. explored the microbiological features of the salivary microbiota to distinguish salivary microbial communities of PSC patients from healthy controls (HC) and ulcerative colitis (UC) patients, by implementing an RF algorithm on meta-genomics data (Iwasawa et al., 2018). Their study highlighted the potential of salivary microbiota as a biomarker to distinguish between PSC patients, UC patients and healthy controls. Their algorithm achieved an AUROC of 0.8756 when distinguishing between PSC and UC, and an AUROC of 0.7423 when distinguishing between PSC and healthy controls (Iwasawa et al., 2018).

### AI and liver echinococcosis

A study by Peng et al. used transcriptomics to generate a predictive model for liver echinococcosis (Peng et al., 2021). Using microarray profiling, the authors identified 1,152 differentially expressed genes. These were selected for Protein-protein interaction (PPI) network analysis to reveal the most critical genes by centrality score, then a RF model was constructed based on hub genes. A particular RF model based on FCGR2B and CTLA4 genes reliably predicted liver hydatid disease with an AUROC of 0.921.

### Liver diseases (general)

One study applied ML to metabolomics to distinguish between HCC and other liver diseases: cirrhosis, and hepatitis (Lin et al., 2011). Using a Feature Support Vector Machine (F-SVM) with selection of 22 metabolites they differentiated the latter two with an accuracy of 89.23 +/− 3.82%, suggesting that metabolites could be used in prediction of liver diseases.

### Limitations

AI models are hard to be understood by humans, they lack in interpretation and considered black boxes. To overcome this important issue in highly regulated areas, such as healthcare, explainable AI (XAI) methods have been developed, based on Shapley values (Shapley; Bussmann et al., 2020). Such models, however, interpret mostly the importance of local features and the contribution of each variable within each model, rather than offering a more global view.

Literature on AI applied to high-throughput omics and clinical data from non-malignant liver diseases is still somewhat limited compared to malignant liver diseases. As evident in the greater number of papers published, attention has been heavily given to studies pertaining to HCC. These were excluded from this review to highlight the use of AI in non-malignant liver diseases. Further, we found only one study on AI utilizing molecular data derived from liver transplant recipients, proving a gap of knowledge in the field of post-transplant liver diseases.

### Future directions

The increased availability of omics data from human samples can contribute to the advancement of precision medicine that relies on data for each individual patient, rather than applying a “one-size-fits-all” model, to fine-tune and personalize the diagnosis and risk stratification of patients. Therefore, there is significant potential for the application of AI for precision medicine in general, and in liver diseases in particular. Further efforts should be directed toward improving the predictive ability of these integrated omics-based algorithms built on a wide range of data, clinical and otherwise. Strategies should be made for the implementation, as well as standardization of such practices. For better interpretation of the AI models, a combination of more interpretable local Shapley values with more global explainable AI methods, such as the Shapley-Lorenz XAI (Giudici and Raffinetti, 2021) have been developed. The advantage of this model resides in both predictive accuracy and interpretation metric and should be used in future research involving AI models. Finally, the practicality of working with large omics datasets on a daily basis need to be considered, including the cost needed to collect and process samples, the time required for analysis and the need for trained personnel and an appropriate facility to perform various omics.

## Conclusion

In this mini review, we screened 20 studies that employed AI on clinical and high-throughput molecular data from non-malignant liver diseases. We found that ML or DL algorithms could predict early disease, stratify different stages, and/or predict survival in liver diseases when using a diversity of omics data in addition to clinical information to build specific models. With greater availability of high-throughput molecular data, there is potential to implement AI for precision medicine in hepatology.

## Author contributions

CB and MB: mini review design, writing of manuscript, and writing the final manuscript. CB, CX, MA, and KP: literature search, abstract selection, and editing the manuscript. KP: input into study design. All authors contributed to the article and approved the submitted version.

## Conflict of interest

The authors declare that the research was conducted in the absence of any commercial or financial relationships that could be construed as a potential conflict of interest.

## Publisher's note

All claims expressed in this article are solely those of the authors and do not necessarily represent those of their affiliated organizations, or those of the publisher, the editors and the reviewers. Any product that may be evaluated in this article, or claim that may be made by its manufacturer, is not guaranteed or endorsed by the publisher.
